# Telemedizinische Betreuung und IT-gestützte Verfahren in der Rheumatologie

**DOI:** 10.1007/s00393-021-01098-w

**Published:** 2021-10-07

**Authors:** Rick McCutchan, Philipp Bosch

**Affiliations:** 1 Universitätsklinik für Innere Medizin II, Medizinische Universität Innsbruck, Innsbruck, Österreich; 2 Klinische Abteilung für Rheumatologie und Immunologie, Medizinische Universität Graz, Auenbruggerplatz 15, 8036 Graz, Österreich

**Keywords:** Krankheitsaktivität, Digitale Gesundheitsanwendungen, COVID-19, Patient reported outcomes, Algorithmusbasierte Applikationen, Disease activity, Digital health applications, Patient reported outcomes, COVID-19, Algorithm-based applications

## Abstract

**Hintergrund:**

Die COVID-19-Pandemie, aber auch die immer größere werdende Beanspruchung des Gesundheitssystems führen dazu, dass die Weiterentwicklung von telemedizinischen Angeboten auch in der Rheumatologie in den Vordergrund gerückt ist.

**Fragestellung:**

Welche Evidenz existiert zu telemedizinischen Angeboten in der Rheumatologie?

**Material und Methode:**

Es erfolgt ein narrativer Review zu bestehenden Arbeiten über Telemedizin in der Rheumatologie.

**Ergebnisse:**

Elektronische „patient reported outcomes“ (ePROs) können von PatientInnen von zu Hause aus bestimmt und dem betreuenden Rheumatologen elektronisch geschickt werden. ePROs könnten in Zukunft dabei helfen zu entscheiden, wann eine klinische Visite notwendig ist. Telemedizinische Visiten wurden bereits durchgeführt bei gut eingestellten PatientInnen mit rheumatischen Erkrankungen mit guten Ergebnissen hinsichtlich Sicherheit und Krankheitsverlauf im Vergleich zu konventionellen Face-to-face-Visiten. Telemedizinische Visiten stellen ein interessantes Tool für Terminpriorisierung und Triage dar, wobei automatisierte, algorithmusbasierte Applikationen derzeit für die klinische Routine noch zu ungenau sind. Die Rolle von Smartphone-Applikationen in der Betreuung von PatientInnen mit rheumatischen Erkrankungen ist noch unklar.

**Diskussion:**

Telemedizin stellt eine interessante Option für bestimmte PatientInnengruppen mit rheumatischen Erkrankungen dar. Abgesehen von Forschung an Effektivität und Sicherheit telemedizinischer Maßnahmen, müssen Entscheidungsträger klare Regeln vorgeben, wie Telemedizin eingesetzt werden soll, um dem individuellen Patienten die bestmögliche Behandlung zukommen zu lassen.

## Abstract (en)

**Background:**

The coronavirus disease 2019 (COVID-19) pandemic and also the ever-increasing demands on the healthcare system, have led to a focus on the further development of telemedical services in rheumatology.

**Objective:**

What is the evidence for telemedical services in rheumatology?

**Material and methods:**

Narrative review of existing literature on telemedicine in rheumatology.

**Results:**

Electronic patient reported outcomes (ePROs) can be determined by patients from their home and sent electronically to the rheumatologist. In future, ePROs may help with the decision whether a patient needs to attend the clinic for a visit or the visit can be rescheduled due to remission and well-being. Telemedicine has already been used for well-controlled patients with rheumatic diseases with good results in terms of safety and disease activity compared to conventional face-to-face visits. Telemedicine represents an interesting tool for appointment prioritization and triaging, while automated algorithm-based applications are currently too imprecise for routine clinical use. The role of smartphone applications in the care of patients with rheumatic diseases is still unclear.

**Discussion:**

Telemedicine represents an interesting option for certain patient populations with rheumatic diseases. Apart from research on the effectiveness and safety of telemedical interventions, decision makers need to set clear rules on how telemedicine should be used to provide the best possible care for the individual patient.

Die Begriffe „Telemedizin“ und „remote care“ haben in den letzten Monaten aufgrund der COVID-19-Pandemie einen neuen Stellenwert in der Medizin erhalten. Ein deutlicher Anstieg an Terminverschiebungen und Verzögerungen therapeutischer Maßnahmen, die v. a. während der ersten COVID-19-Infektionswelle auftraten, führten dazu, dass eine adäquate PatientInnenversorgung größtenteils nicht mehr möglich war [5]. Insbesondere im Bereich der Rheumatologie, einem Fach, in dem frühe Diagnosen und Therapien maßgeblich für den Therapieerfolg sind, scheint die Etablierung und Weiterentwicklung telemedizinischer Systeme daher essenziell.

Telemedizin befasst sich mit der Verwendung von Telekommunikation und virtueller Technologie zur Überbrückung von Distanzen bei der PatientInnenbetreuung. Sie bietet hierbei eine Reihe an Möglichkeiten, angefangen von Terminpriorisierung für PatientInnen mit vermeintlich rheumatischen Erkrankungen über diagnostische Erstgespräche bis hin zu kurz- und langfristigen Monitoringvisiten.

Die Nutzung von telemedizinischen Angeboten ist hierbei nicht nur auf Pandemiezeiten beschränkt, sondern bietet auch – neben positiven finanziellen Aspekten – die Möglichkeit einer weitreichenden Versorgung in Gebieten mit geringem Angebot an rheumatologischen Ambulanzen [17]. Die Erfassung und Speicherung von PatientInnendaten auf digitale Art und Weise (über Websites, Smartphone-Apps etc.) hat außerdem den Vorteil der einfachen Schaffung von Registerdatenbanken, mithilfe derer Forschung mit dem Ziel der verbesserten PatientInnenversorgung betrieben werden kann.

Um die telemedizinische PatientInnenversorgung mit dem Goldstandard der F2F(„face-to-face“)-Betreuung zu vergleichen, gilt es zuerst zu verstehen, welche Daten telemedizinisch erhoben werden können.

## Patient reported outcomes

Um die Krankheitsaktivität nicht nur anhand von radiologischen Gelenkveränderungen, geschwollenen und schmerzhaften Gelenken, sondern auch anhand der subjektiven Einschätzung der PatientInnen bewerten zu können, wurden bereits in den 1970er-Jahren die ersten „patient reported outcomes“ (ePROs) entwickelt. Das Ziel dieser war es, die Gesamtheit der Faktoren zu evaluieren, die den Leidensdruck und die alltägliche Funktion bei Menschen mit einer chronischen Erkrankung ausmachen, und durch ihre Erfassung bessere Therapieziele zu formulieren. Zwei der ersten PROs waren der Health Assessment Questionnaire (HAQ) [7] sowie die Arthritis Impact Measurement Scale (AIMS) [19]. Der HAQ versucht das alltägliche Leben mithilfe von Angaben zur Fähigkeit, sich anzuziehen, aufzustehen, zu essen, Körperpflege zu betreiben etc., zu erfassen. Mobilität, soziale Funktion und Aktivität, körperliche und Alltagsaktivität, Schmerzen, Depressionen, Ängste und Geschicklichkeit sind die 9 Hauptgruppen der AIMS, deren Fragenpool aus insgesamt 46 Fragen besteht.

Nach einigen Modifikationen und Erneuerungen dieser Tests haben sich das Feld der PROs und die Anzahl der Messmöglichkeiten rasant erweitert. In einem Review von 2011 waren es 250 verschiedene Messmöglichkeiten [14], wobei die European Alliance of Associations for Rheumatology (EULAR) sogar eine eigene Website betreut, die die verschiedenen Outcome-Parameter in multiplen Sprachen auflistet und aktualisiert [11].

Durch die fortschreitende Digitalisierung werden PRO-Papierfragebögen kontinuierlich von elektronisch erfassten „patient reported outcomes“ (ePROs) abgelöst. Vermeidung von Übertragungsfehlern, verringerte administrative Tätigkeiten und damit verbundene Kosteneinsparungen sind nur einige potenzielle Vorteile von ePROs [3]. Studien zeigten des Weiteren, dass die elektronische Erfassung von PROs die gleichen Ergebnisse bringt wie die herkömmliche Erfassung mittels Papierfragebögen [35].

Durch die fortschreitende Digitalisierung werden PRO-Papierfragebögen von ePROs abgelöst

Während PRO-Papierfragebögen in erster Linie direkt in den Ambulanzen und Warteräumen („on-site“) ausgefüllt werden, ermöglichen ePROs die Datenerfassung und Datensendung aus den eigenen 4 Wänden („off-site“). Man könnte sogar argumentieren, dass aufgrund der vertrauten Umgebung die hierbei erhobenen Daten die eigentliche Krankheitsaktivität genauer widerspiegeln als Daten, die in einer medizinischen Ambulanz angegeben werden (ähnlich dem White-Coat-Syndrom bei der arteriellen Hypertonie) [25].

In Bezug auf das Gerät, mit dem die digitale Datenerfassung erfolgt, ist das BYOD(„bring your own device“)-Konzept auf dem Vormarsch. Der konventionelle Weg war bisher, den PatientInnen ein Gerät im Rahmen der Datenerhebung (meist in den Kliniken, also „on-site“) zur Verfügung zu stellen. Diese Herangehensweise ist mit einigen Schwachpunkten vergesellschaftet, die Kosten, Versorgungsangebot, Einarbeitung des betreuenden Personals und die Erhaltung des Geräts beinhalten. Aufgrund der nun alltäglichen und breiten Nutzung von Computern und Smartphones ist daher anzunehmen, dass die Datenerfassung in Zukunft immer mehr auf den eigenen elektronischen Geräten erfolgen wird [3].

## Elektronisch erfasste „patient reported outcomes“ und Datenerhebung in der Rheumatologie

(e)PROs wie der „Routine Assessment of Patient Index Data 3“(RAPID-3)- oder der „Rheumatoid Arthritis Impact of Disease“(RAID)-Fragebogen werden bereits seit Längerem in der klinischen Routine verwendet, um sich eine bessere Vorstellung von dem subjektiven Krankheitserleben der individuellen PatientInnen machen zu können [10, 24]. Diese (e)PROs können von PatientInnen prinzipiell problemlos digital ausgefüllt und den betreuenden ÄrztInnen vor einer Visite zugeschickt werden [31].

**Figure 1:**
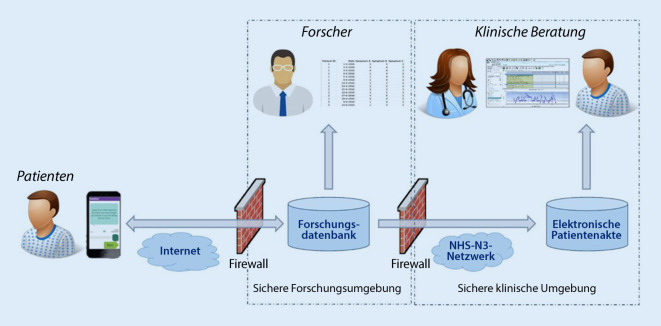

Ähnliches gilt für Laborparameter, die im Vorhinein bei HausärztInnen oder bei einem Institut abgenommen werden können. Schwieriger wird es bei der körperlichen Untersuchung. Während Gelenkschmerzen von PatientInnen recht gut lokalisiert werden können, insbesondere wenn sie sich in einem Zustand der geringen Krankheitsaktivität befinden, ist die Einschätzung einer Gelenkschwellungen oftmals unterschiedlich von der von RheumatologInnen [2, 27]. Die British Society of Rheumatology hat nichtsdestotrotz ein Video veröffentlicht, in dem PatientInnen beigebracht wird, einen Gelenkstatus selbst durchzuführen [13] mit dem Ziel der Sammlung dieser Informationen über eine Smartphone-App in eine elektronische Krankenakte (Abb. 1; [1]).

PatientInnen, die nach einer Schulung in der Lage sind, einen adäquaten Gelenkstatus durchzuführen, wären ideale KandidatInnen für telemedizinische Visiten. Krankheitsaktivitätsscores wie der „Clinical Disease Activity Index“ (cDAI) könnten so berechnet und den RheumatologInnen im Vorhinein zur Verfügung gestellt werden. Die alleinige Verwendung von ePROs hingegen könnte in Zukunft den Nutzen bringen zu evaluieren, ob eine F2F-Visite überhaupt notwendig ist. Größere Studien hierzu werden jedoch noch benötigt.

Die Bereitschaft von PatientInnen, ihre Daten kontinuierlich und konsequent einzugeben, ist durchaus gegeben, vorausgesetzt dieser folgt eine Reaktion des/der betreuenden RheumatologIn, wenn Werte einer höheren Krankheitsaktivität entsprechen [20]. Generell scheint jedoch noch eine Skepsis gegenüber telemedizinischen Maßnahmen zu bestehen, wie eine rezente Umfrage aus Spanien zeigt. Nur 52,7 % der befragten PatientInnen mit verschiedenen rheumatischen Erkrankungen gaben an, dass eine Telefonvisite nützlich zum Monitoring ihrer Krankheit wäre, wobei auch nur 37,9 % angaben, bereits eine solche Visite gehabt zu haben [18].

## Studien zur Telemedizin in der Rheumatologie

### „Remote monitoring“

Die wohl einfachste Form des „remote monitoring“ stellt die Telefonie dar. Über 4 Mrd. Menschen weltweit haben die Möglichkeit, ein Telefon zu benutzen [12], was sich auch darin ausdrückt, dass die Telefonsprechstunde die meistgenutzte Alternative zur F2F-Visite ist [33].

In einer randomisiert kontrollierten Studie (RCT) aus dem Jahr 2018 mit 294 PatientInnen mit rheumatoider Arthritis, konnte gezeigt werden, dass eine telefonische Visite einer F2F-Visite nicht unterlegen ist, wenn man als primären Endpunkt den DAS(Disease Activity Score)28 nach 52 Wochen betrachtet. Insgesamt verringerte sich die Anzahl der F2F-Visiten in dieser Studie um das 4‑Fache (1,75 [Standardabweichung/SD 1,03] Visiten/Jahr vs. 4,15 [SD 1,0] Visiten/Jahr), wobei auch gezeigt werden konnte, dass die telefonische Visite von Krankenpflegepersonal durchgeführt werden konnte ohne Unterschiede im DAS28, Anzahl an Rezidiven oder Anzahl der F2F-Visiten im Vergleich zu Visiten, die von ÄrztInnen geführt wurden [4].

Telefonvisiten können F2F Visiten ergänzen

Telemedizin ist jedoch nicht zwangsläufig nur für PatientInnen mit gut eingestellter Krankheitsaktivität eine Option. Pers et al. untersuchten den Nutzen einer Smartphone-Applikation bei PatientInnen mit rheumatoider Arthritis (RA) in moderater/hoher Krankheitsaktivität, bei denen eine Therapie mit einem „disease-modifying anti-rheumatic drug“ (DMARD) eingeleitet wurde [23]. In dieser RCT wurde die erste Hälfte der PatientInnen 1‑mal wöchentlich per E‑Mail darauf hingewiesen, einige ePROs inklusive RAPID‑3 und auto-DAS28 in eine Smartphone-App zu notieren [26]. War der RAPID-3 > 12/30 Punkten oder wurden nach 2 Monaten kaum Daten von PatientInnen eingegeben, wurden diese von einem „clinical case manager“ telefonisch kontaktiert, um einen F2F- oder telefonischen Termin mit einer RheumatologIn auszumachen. In der zweiten Gruppe erfolgten routinemäßige F2F-Kontrollen nach Einschätzung der betreuenden RheumatologIn. Nach 6 Monaten fanden sich keine Unterschiede zwischen den Gruppen anhand von DAS28, RAPID‑3 oder HAQ. In der Smartphone-App-Gruppe war die Anzahl an F2F-Visiten deutlich geringer als in der Vergleichsgruppe (0,42 [SD 0,58] vs. 1,93 [SD 0,55]; *p* < 0,05), während die Anzahl an Telefonaten deutlich höher war (2,67 [SD 2,58] vs. 0,41 [SD 1,19]; *p* < 0,01).

Gerade in Situationen, in denen Distanz eine entscheidende Rolle spielt, erscheint der Einsatz von Telemedizin sinnvoll. In einer 2017 veröffentlichten RCT konnte gezeigt werden, dass RA-PatientInnen (*n* = 85) aus dem ländlichen Raum, die eine 3‑monatliche Telekonferenz mit einer RheumatologIn hatten, ähnliche Ergebnisse bei DAS28-CRP (Disease activity score 28 - C‑reactive protein), RADAI (Rheumatoid Arthritis Disease Activity Index), HAQ, sowie Lebensqualität und Zufriedenheit hatten im Vergleich zu einer Gruppe, die routinemäßige F2F-Visiten hatte. Hierbei erfolgten die klinische Untersuchung sowie die Unterstützung bei der Telekonferenz durch eine/n PhysiotherapeutIn [32].

Auch eine rezente chinesische Studie konnte zeigen, dass die Integrierung von Telemedizin gemeinsam mit F2F-Visiten eine Optimierung der Versorgung von PatientInnen mit rheumatischen Erkrankungen darstellen könnte [30], wobei derzeit multiple Studien zu dem Thema in Arbeit sind (Tab. 1).

**Table 1:** 

Name der Studie	Erkrankungen	Studien-ID
Comparing Modes of Telehealth Delivery: Phone vs. Video Visits (ASSISt)	Rheumatische Erkrankungen	NCT04616118
Effectiveness of Tele-rheumatology for Delivering High Quality Rheumatology Care During the COVID-19 Crisis (EVOLVE)	Rheumatische Erkrankungen	NCT04704544
Telehealth Stretching Exercise Program for Women with Fibromyalgia During the Covid-19 Pandemic	Fibromyalgie	NCT04690400
Telehealth CBT for Adolescents and Young Adults with Childhood-onset Systemic Lupus Erythematosus (cSLE)	cSLE	NCT04335643
Evaluation of a Non-face to Face Multidisciplinary Health Care Model in a Population with Rheumatoid Arthritis	RA	NCT04768413
E‑learning in Patient Education to Patients with Rheumatoid Arthritis (WEB-RA)	RA	NCT04669340
Pilot Study of the Impact of Giant Cell Arteritis and Its Treatment on the Autonomy of the Elderly in the First Year of Care (EPACAPA)	GCA	NCT03961113
Impact of a Hybrid Medical Care Model in the Rheumatoid Arthritis Patient-reported-outcomes Measures: A Non-inferiority Study	RA	NCT04558905
The Effects of Tele-Yoga in Ankylosing Spondylitis Patients	axSpA	NCT04803383
Monitoring Spondyloarthritis With SpA-Net (TeleSpA)	SpA	NCT04673825
Impact of Barriers and Facilitators to Physical Activity in Patients with Inflammatory Arthritis (ImBAIA)	Entzündliche Arthritis	NCT04426747
A Novel Mobile App & Population Management System to Manage Rheumatoid Arthritis Flares	RA	NCT02822521
A Multi-Center Clinical Trial to Determine the Impact of a Mobile Health Application on Rheumatoid Arthritis Shared Decision Making	RA	NCT03773471
Multidisciplinary Approach for Treat To Target In Rheumatoid Arthritis (MAPPIT-RA)	RA	NCT02720874
Walk for Rheumatoid Arthritis (WARA Study)	RA	NCT02467205

*axSpA* axiale Spondyloarthritis, *cSLE* juveniler systemischer Lupus erythematodes, *GCA* Riesenzellarteriitis, *RA* rheumatoide Arthritis, *SpA* Spondyloarthritis

### PatientInnenpriorisierung und Diagnostik

**Figure 2:**
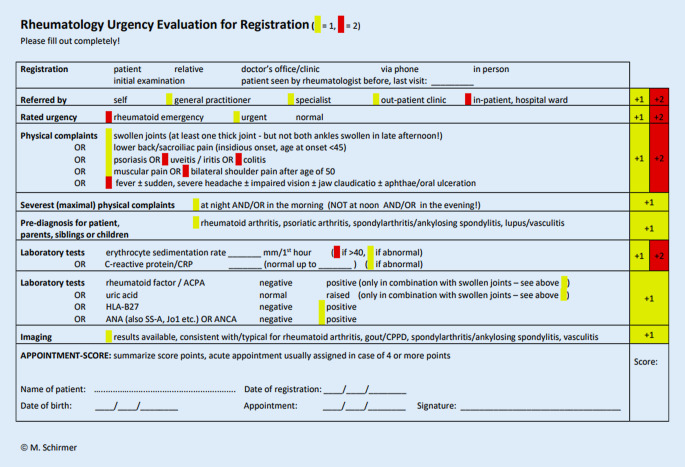

In rheumatologischen Ambulanzen finden sich nicht selten PatientInnen mit muskuloskeletalen Beschwerden, die in erster Linie eine orthopädische und nicht rheumatologische Grunderkrankungen vorweisen. PatientInnenpriorisierung und Triage erfolgt bereits an vielen Zentren auf unterschiedliche Art und Weise. In der Rheumatologie-Ambulanz der Medizinischen Universität Innsbruck wird beispielsweise der „rheumatology urgency score“ (RUS) verwendet, um die Dringlichkeit eines Termins einzuschätzen [8]. Ein dringlicher Fall wird hierbei bei einem Punktescore von ≥ 4 definiert (Abb. 2).

Legget et al. veröffentlichten 2001 Ergebnisse einer Kohortenstudie, in der 100 PatientInnen mit dem Verdacht einer rheumatischen Erkrankung eingeschlossen wurden. Es fand eine rein telefonische Kontaktaufnahme zwischen PatientInnen, HausärztInnen (= ÜberweiserInnen) und RheumatologInnen statt, wobei sich die PatientInnen dabei in der HausärztInnen-Praxis aufhielten. Nach einer initialen Anamnese und Befundbesprechung wurde auch eine Videokamera eingeschaltet. Die RheumatologInnen stellten sowohl nach dem Telefongespräch als auch nach der Videotelefonie eine Verdachtsdiagnose. Die PatientInnen hatten schließlich einen F2F-Termin in der rheumatologischen Ambulanz, wo die finale Diagnose gestellt wurde (= Goldstandard). Die diagnostische Genauigkeit nach dem Telefongespräch war 71 %, während jene nach der Videotelefonie 97 % ausmachte [16]. Ähnliche Ergebnisse wurden auch aus einer anderen Kohortenstudie berichtet, bei der die diagnostische Genauigkeit nach einer Videokonferenz bei 79 % lag [21].

Wenngleich diese Ergebnisse Erfolg versprechend klingen, fehlen größere Studien, welche die diagnostische Genauigkeit von telemedizinischen Untersuchungen evaluieren. Online-Tools und Smartphone-Applikationen, die nach Eingabe von Symptomen und Befunden dabei helfen, zwischen einer entzündlich rheumatischen Krankheit und einer anderen Ursache für die Beschwerden zu differenzieren, sind derzeit noch in Erprobung/Entwicklung bzw. zu ungenau für eine routinemäßige Verwendung in der Praxis [15].

### Smartphone-Applikationen als Monitoringhilfe

Da digitale Gesundheitsanwendungen (DiGA) in Deutschland nun in aller Munde sind und die ersten Applikationen zur supportiven Behandlung von PatientInnen mit rheumatischen Erkrankungen schon in den Startlöchern stehen, stellt sich die Frage, inwieweit die Verwendung dieser Programme PatientInnen von Nutzen sein kann.

Eine 2016 veröffentlichte Studie von Salaffi et al. zeigte [29], dass mittels intensiver telemedizinischer Überwachung mehr RA-PatientInnen eine Krankheitsremission (cDAI ≤ 2,8) nach 1 Jahr erreichten als jene, die routinemäßige Kontrollen hatten (38,1 % vs. 25 %, *p* < 0,01). Das Studiendesign dieser RCT umfasste 5 F2F-Visiten in beiden Gruppen und zusätzliche 8 telemedizinische Visiten in der Interventionsgruppe. In diesen telemedizinischen Visiten wurde anhand des RADAI bestimmt, ob die Notwendigkeit einer Therapieadaptierung gegeben war. Beim Ausbleiben einer Besserung wurde die Basistherapie entsprechend einem vorgegebenen Schema adaptiert [34]. In der Kontrollgruppe erfolgte die Therapieadaptierung individuell nach Einschätzung der betreuenden RheumatologInnen.

Eine weitere Gruppe der Smartphone-Applikationen befasst sich mit Symptomtracking: Schrittzähler und Aktivitätsuhren haben nicht nur das Potenzial, durch Motivation die physische Aktivität zu steigern [28], sondern könnten laut einer laufenden RCT in Zukunft auch dabei helfen, Rückschlüsse auf die Krankheitsaktivität zu ziehen. Durch maschinelles Lernen von Daten aus Schrittzählern konnte mit hoher Sensitivität (95,7 %) und Spezifität (96,7 %) festgestellt werden, ob bei PatientInnen mit RA oder Spondyloarthritis ein Krankheitsschub vorlag oder nicht [9]. Ähnliche Studien fanden auch Zusammenhänge zwischen Krankheitsaktivität bei RA-PatientInnen (DAS28) und unüblichen Outcomes wie der Händegreiffunktion (gemessen mittels handyverbundenem Dynamometer) [6] sowie der Beschleunigung beim Gehen [22].

## Zukunftsperspektiven und ethische Überlegungen

Die COVID-19-Pandemie hat den Startschuss für den Aufbau und die Weiterentwicklung der telemedizinischen Betreuung in der Rheumatologie gegeben. Laufende Studien zu dem Thema finden sich in Hülle und Fülle (s. Tab. 1) und befassen sich unter anderem mit der Videotelefonie als Alternative zur F2F-Visite, als Option zur PatientInnenschulung und Anleitung von körperlichen Übungen sowie als Plattform für kognitive Verhaltenstherapie. Außerdem werden nun nach und nach Smartphone-Applikationen und Programme für Patientinnen mit rheumatischen Krankheiten präsentiert, die das Ziel verfolgen, durch Eingabe von Symptomen und subjektiven Krankheitszuständen die PatientInnen in die eigene Behandlung besser einzubinden und die Möglichkeit einer telemedizinischen Betreuung zu erleichtern. Gerade im Hinblick auf die Möglichkeit, DiGA zu verschreiben und zu verrechnen, kann man annehmen, dass in den nächsten Jahren eine Vielzahl neuer Gesundheitsapplikationen auf den Markt kommen wird.

Telemedizin sollte als zusätzliche Option und nicht als alleinige Behandlungsmaßnahme verstanden werden

Wie die Telemedizin die PatientInnenbetreuung in der Rheumatologie in Zukunft beeinflussen oder gar revolutionieren wird, ist noch unklar. Ein EULAR-Projekt, das zu diesem Thema „points to consider“ entwickelt, ist derzeit in Arbeit und wurde bereits beim EULAR Meeting im Juni 2021 präsentiert. Hier wurden auch die Herausforderungen und ethischen Überlegungen einer telemedizinischen Betreuung diskutiert. Telemedizin sollte für und mit PatientInnen mit rheumatischen Erkrankungen entwickelt werden, wobei im Vordergrund die Verbesserung und Erleichterung der PatientInnenbetreuung stehen muss. Trotz Ausbaus telemedizinischer Maßnahmen muss für die PatientInnen weiterhin die Möglichkeit einer konventionellen F2F-Visite auf Wunsch möglich sein. Telemedizin sollte daher als zusätzliche Option und nicht als alleinige Behandlungsmaßnahme verstanden werden. Der adäquate Umgang mit PatientInnendaten steht dabei ebenso im Fokus wie die individualisierte Betreuung von PatientInnen mit rheumatischen Erkrankungen.

## Fazit für die Praxis

- Elektronische „patient reported outcomes“ (ePROs) bieten langfristige Vorteile im Vergleich zu PROs, die über Papierfragebögen abgefragt werden.
- ePROs haben das Potenzial festzustellen, ob eine klinische Visite bei PatientInnen mit rheumatischen Erkrankungen notwendig ist oder nicht. Größere Studien hierzu fehlen jedoch noch.
- Telemedizinische Visiten wurden zum Monitoring von PatientInnen mit rheumatischen Erkrankungen bereits verwendet, und erste Studien zeigen gute Ergebnisse im Hinblick auf Sicherheit und Krankheitsverlauf.
- Telemedizinische Visiten stellen ein interessantes Tool für Terminpriorisierung und Triage dar, wobei automatisierte, algorithmusbasierte Applikationen derzeit für die klinische Routine noch zu ungenau sind.
- Trotz erster Erfolg versprechender Ergebnisse ist der Wert von Smartphone-Applikationen für die Behandlung und das Monitoring von PatientInnen mit rheumatischen Erkrankungen noch unklar.
- Die Option einer telemedizinischen Visite darf PatientInnen nicht die Möglichkeit nehmen, eine konventionelle Face-to-face-Visite in Anspruch zu nehmen.

## References

[1] Austin L, Sharp CA, Van Der VSN (2020). Providing „the bigger picture“: benefits and feasibility of integrating remote monitoring from smartphones into the electronic health record: findings from the remote monitoring of rheumatoid arthritis (REMORA) study. Baillieres Clin Rheumatol.

[2] Cheung PP, Ruyssen-Witrand A, Gossec L (2010). Reliability of patient self-evaluation of swollen and tender joints in rheumatoid arthritis: a comparison study with ultrasonography, physician, and nurse assessments. Arthritis Care Res.

[3] Coons SJ, Eremenco S, Lundy JJ (2015). Capturing patient-reported outcome (PRO) data electronically: the past, present, and promise of ePRO measurement in clinical trials. Patient.

[4] De Thurah A, Stengaard-Pedersen K, Axelsen M (2018). Tele-health followup strategy for tight control of disease activity in rheumatoid arthritis: results of a randomized controlled trial. Arthritis Care Res.

[5] Dejaco C, Alunno A, Bijlsma JW (2020). Influence of COVID-19 pandemic on decisions for the management of people with inflammatory rheumatic and musculoskeletal diseases: a survey among EULAR countries. Ann Rheum Dis.

[6] Espinoza F, Le Blay P, Coulon D (2016). Handgrip strength measured by a dynamometer connected to a smartphone: a new applied health technology solution for the self-assessment of rheumatoid arthritis disease activity. Baillieres Clin Rheumatol.

[7] Fries JF, Spitz P, Kraines RG (1980). Measurement of patient outcome in arthritis. Arthritis Rheum.

[8] Ghazal MO, Schirmer M (2017). Proposal for an urgency score as general referral strategy to second-care rheumatology. Ann Musculoskelet Med.

[9] Gossec L, Guyard F, Leroy D (2019). Detection of flares by decrease in physical activity, collected using wearable activity trackers in rheumatoid arthritis or axial spondyloarthritis: an application of machine learning analyses in rheumatology. Arthritis Care Res.

[10] Gossec L, Paternotte S, Aanerud GJ (2011). Finalisation and validation of the rheumatoid arthritis impact of disease score, a patient-derived composite measure of impact of rheumatoid arthritis: a EULAR initiative. Ann Rheum Dis.

[11] http://Oml.Eular.Org/Index.Cfm. Zugegriffen: 9. Juni 2021

[12] https://Www.Statista.Com/Statistics/274774/Forecast-of-Mobile-Phone-Users-Worldwide/. Zugegriffen: 9. Juni 2021

[13] https://Www.Youtube.Com/Watch?V=Sbsjkmynoaw&T=248s. Zugegriffen: 10. Juni 2021

[14] Katz PP (2011). Introduction to special issue: patient outcomes in rheumatology, 2011. Arthritis Care Res.

[15] Knitza J, Mohn J, Bergmann C (2021). Accuracy, patient-perceived usability, and acceptance of two symptom checkers (Ada and Rheport) in rheumatology: interim results from a randomized controlled crossover trial. Arthritis Res Ther.

[16] Leggett P, Graham L, Steele K (2001). Telerheumatology—diagnostic accuracy and acceptability to patient, specialist, and general practitioner. Br J Gen Pract.

[17] Lewtas J (2001). Telemedicine in rheumatology. J Rheumatol.

[18] López-Medina C, Escudero A, Collantes-Estevez E (2020). COVID-19 pandemic: an opportunity to assess the utility of telemedicine in patients with rheumatic diseases. Ann Rheum Dis.

[19] Meenan RF, Gertman PM, Mason JH (1982). The arthritis impact measurement scales. further investigations of a health status measure. Arthritis Rheum.

[20] Navarro-Millán I, Zinski A, Shurbaji S (2019). Perspectives of rheumatoid arthritis patients on electronic communication and patient-reported outcome data collection: a qualitative study. Arthritis Care Res.

[21] Nguyen-Oghalai TU, Hunter K, Lyon M (2018). Telerheumatology: the VA experience. South Med J.

[22] Nishiguchi S, Ito H, Yamada M (2014). Self-assessment tool of disease activity of rheumatoid arthritis by using a smartphone application. Telemed J E Health.

[23] Pers YM, Valsecchi V, Mura T (2020). A randomized prospective open-label controlled trial comparing the performance of a connected monitoring interface versus physical routine monitoring in patients with rheumatoid arthritis. Rheumatology.

[24] Pincus T, Yazici Y, Bergman MJ (2009). RAPID3, an index to assess and monitor patients with rheumatoid arthritis, without formal joint counts: similar results to DAS28 and CDAI in clinical trials and clinical care. Rheum Dis Clin North Am.

[25] Pioli MR, Ritter AM, De Faria AP (2018). White coat syndrome and its variations: differences and clinical impact. Integr Blood Press Control.

[26] Potter T, Wild A, Edwards K (2006). Patients’ own ability to assess activity of their rheumatoid arthritis. Baillieres Clin Rheumatol.

[27] Radner H, Grisar J, Smolen JS (2012). Value of self-performed joint counts in rheumatoid arthritis patients near remission. Arthritis Res Ther.

[28] Ridgers ND, Timperio A, Brown H (2017). A cluster-randomised controlled trial to promote physical activity in adolescents: the Raising Awareness of Physical Activity (RAW-PA) Study. BMC Public Health.

[29] Salaffi F, Carotti M, Ciapetti A (2016). Effectiveness of a telemonitoring intensive strategy in early rheumatoid arthritis: comparison with the conventional management approach. BMC Musculoskelet Disord.

[30] So H, Chow E, Cheng I, Lau X, Li T, Szeto C, Tam L (2021) Use of Telemedicine for Follow-up of Lupus Nephritis in the COVID-19 Outbreak: The 6-month Results of a Randomized Controlled Trial [abstract]. Arthritis Rheumatol 73(suppl 10) 10.1177/09612033221084515 PMC8902321 35254169

[31] Solomon DH, Rudin RS (2020). Digital health technologies: opportunities and challenges in rheumatology. Nat Rev Rheumatol.

[32] Taylor-Gjevre R, Nair B, Bath B (2018). Addressing rural and remote access disparities for patients with inflammatory arthritis through video-conferencing and innovative inter-professional care models. Musculoskelet Care.

[33] Thwaites C, Ryan S, Hassell A (2008). A survey of rheumatology nurse specialists providing telephone helpline advice within England and Wales. Rheumatology.

[34] Verstappen SM, Jacobs JW, Van Der Veen MJ (2007). Intensive treatment with methotrexate in early rheumatoid arthritis: aiming for remission. Computer Assisted Management in Early Rheumatoid Arthritis (CAMERA, an open-label strategy trial). Ann Rheum Dis.

[35] Zbrozek A, Hebert J, Gogates G (2013). Validation of electronic systems to collect patient-reported outcome (PRO) data-recommendations for clinical trial teams: report of the ISPOR ePRO systems validation good research practices task force. Value Health.

